# Youth perceptions of urban waterfront environments for stress relief: a social media text analysis study in Beijing

**DOI:** 10.3389/fpubh.2026.1816233

**Published:** 2026-04-07

**Authors:** Zecheng Li, Norhuzailin Binti Hussain, Mohd Yazid Mohd Yunos

**Affiliations:** Faculty of Design and Architecture, Universiti Putra Malaysia, Serdang, Selangor, Malaysia

**Keywords:** seasonal differences, social media analysis, stress recovery, urban blue space, youth

## Abstract

Urbanization has aggravated the mental health problems of youth. Blue spaces have been recognized as potential non-pharmacological intervention resources. However, there is a lack of literature focusing on seasonal water body variations in northern cities and youth populations. Using a collection of 4,502 social media posts and entries on Douyin and Xiaohongshu with a geographical location, this study used three-level NVivo thematic coding and triangulation to systematically analyze how the youth in Beijing perceive the stress-relief function of urban waterfronts. The findings show that waterfront areas are superior in terms of sensory experience compared to non-waterfront areas, with a 3.05-fold difference. The distinct waterfront value of calmness that exists in waterfront areas is completely absent in non-waterfront areas. Winter water bodies in northern cities exhibit “alternative restoration” characteristics, where frozen landscapes and seasonal rituals provide distinct pathways for psychological adjustment. The ice landscape at 5.8% and seasonal rituals at 3.9% reflect the alternative pathways for psychological adjustment. The five types of water bodies constitute a differentiated functional spectrum of “tranquil aesthetics” to “social sharing.” The natural ones are mainly sensory and emotional restoration while the artificial ones are mainly social and accessibility. This study affirms the relevance of Attention Restoration Theory and Stress Recovery Theory in the Chinese urban blue space context. It also enriches the seasonal dimension of the restorative environment theory through the proposed “alternative restoration” hypothesis. Further, this research fills in the gap of youth-centric research. Finally, it provides empirical evidence for refined planning of youth-friendly waterfront spaces.

## 1 Introduction

Rapid urbanization has changed the demographics and lifestyles of the Chinese people. The rising mental health issues have become a focal point of attention from academia and public policy. In huge cities, young people bear the combined stress of exam competition, job pressure, housing affordability and societal expectations. According to a nationwide large-scale survey, the psychological distress of Chinese youth is not merely the result of individual factors. In fact, the mental health status of youth is determined by multi-level influences, namely individual, school and provincial level factors (1). Given the rise in mental health concerns amongst young people, natural environments are increasingly being seen as a non-drug intervention. A study conducted in three cities in China showed that young adults’ health perceptions of urban green spaces have strong emotional effects. This shows that contact with nature can help improve the psychological wellbeing of young people living in cities (2). Urban water bodies, often termed as blue spaces, are unique natural environments. Because of their distinctive sensory experience and positive psychological impact, researchers are increasingly using them to study health and the environment. Over the past decade, a significant amount of evidence from countries around the world has emerged. Comprehensive systematic reviews have explored the various alleged health benefits of blue spaces. As a result of these reviews, researchers identified exposure to water through a range of mechanisms (some of which overlap) offers health benefits. Some of these mechanisms include greater physical activity, better social interaction, cleaner air and psychological restoration (3). Subsequent systematic reviews of blue space interventions further confirm that the positive effects of aquatic environments on health and wellbeing are cross-cultural (4). Following the systematic review and meta-analysis, researchers have identified a multitude of mechanistic pathways that link blue spaces to health; these include attention restoration, stress reduction, facilitation of physical activity and increased social cohesion (5). According to research conducted in 18 countries, green spaces and blue spaces have a positive association with mental health outcomes, which vary by culture (6). Further supporting the evidence base for health benefits associated with aquatic environments, quantitative systematic reviews and meta-analyses specifically focused upon urban blue spaces (7). The most recent umbrella review synthesising epidemiological meta-analyses on green and blue spaces suggests this field is advancing toward more nuanced and mechanism-oriented research (8).

According to the Attention Restoration Theory (ART) and the Stress Recovery Theory (SRT), natural environments have psychological benefits. ART contends that natural environments have four restorative qualities (i.e., being away, extent, fascination, compatibility) that help people replenish cognitive resources depleted through prolonged directed attention.

Comprehensive reviews have traced the theoretical evolution of ART and design applications to the restorative environment. The theory’s guiding power is underscored by the understanding of environment–psychology interactions (9). According to recent scholarship, nature might help by changing the way our goals work. In this way, the picture of ART has been enriched (10). In contrast to ART’s focus on restoration of cognitive function, SRT stresses the restorative influence of nature on physiological stress response. As per this theory, nature scenes can counter stress by evoking positive feelings and lowering sympathetic nervous responses. According to meta-analyses that integrated empirical studies on the relationship between physiological stress response and the natural environment, contact with nature is stress-reducing from both biophilia and stress recovery perspectives (11). Study after study finds that spending time in nature is incredibly beneficial for mental health. Metaanalytic work on adults with mental health issues confirms that being in nature has lasting positive effects on people’s wellbeing (12). These two theories give strong ideas for understanding how waterfront environments help people in the present study. Among different types of space, it has been found that the aquatic environment has restorative value of a different kind from green spaces. The water surface spectacle, the sound of flowing water and the microclimate at waterfront sites are what make aquatic places attractive. The findings of a study that utilized population-level data in a retrospective manner indicated that urban blue spaces may potentially mitigate the impact of socioeconomic deprivation on mental health, which may then present significant opportunities for aquatic assets to enhance health equity (13).

Most conventional environmental perception research has adopted questionnaire surveys and experimental observations. However, these methods demonstrate some limitations, such as limited sample size, spatiotemporal coverage, and ecological validity. Due to the popularity of social media, researchers are getting fresh data and new methodologies for environmental perception research. The use of geolocated Twitter data to assess the difference between sentiment of people in parks and that of people elsewhere in New York City showed that sentiments expressed on social media are effective measures of wellbeing and that there is significant spatial heterogeneity in the affect associated with parks (14). The method of this study which integrates social media data with place, location and sentiment analysis, offers an important reference for further study. Approaches within Social Media have proved advantageous for cross-cultural and fine-grained analyses. Using online reviews and natural language processing techniques in cross-cultural comparative research, environmental features influencing positive and negative perception of urban parks were identified. The work illustrates the potential of such methods for extracting granular information on environmental perception (15). Various studies using geotagged social media data to analyze spatiotemporal patterns of urban activities and differences between demographic groups provide methodological insights on studying environmental use behavior across populations (16). These investigations collectively indicate that social media text analysis can compensate for limitations inherent in traditional methods and is particularly well-suited for examining environmental perceptions among youth, who represent heavy users of social media platforms.

Douyin and Xiaohongshu were selected as primary data sources based on three methodological considerations. First, these platforms demonstrate high penetration rates among Chinese youth aged 18–35, representing the dominant social media ecosystem for this demographic cohort. Second, both platforms feature robust geotagging functionalities that enable precise spatial attribution of user-generated content. Third, the visual-centric and “check-in” culture prevalent on these platforms captures spontaneous environmental experiences, as users share waterfront visits voluntarily rather than in response to survey instruments, thereby minimizing research-induced response bias (17).

A lot has been done in researching blue space health benefits yet, the literature displays some gaps. Most studies focus on temperate oceanic or subtropical regions, while seasonal variations of water bodies in northern cities have been largely overlooked. According to research on winter urban park “white spaces,” northern cities’ water bodies take on very different physical forms during winter, implying their restorative characteristics warrant reconsideration (18). At the same time, we are limited in research that differentiates water types. Earlier an attempt was made to understand the relationship between freshwater types and health benefits (19). Moreover, scholars have argued that the emphasis on freshwater blue spaces (e.g., lakes, rivers, wetlands) represents a vital research agenda (20). However, very few studies compared across the different freshwater water body type categories (i.e., lakes, wetlands, rivers, park water systems, urban water features). Furthermore, young people, as both significant bearers of urban stress and primary users of social media, have yet to receive adequate attention in blue space perception research. The deep learning-based research of social media images from central Beijing has revealed features of youth’s perception of the cultural ecosystem service and offers methodological insights into youth-centric blue-space studies (21). Research studies of feelings involving four seasons in rivers and lakes of blue space in inland Chinese cites, have just started to address the interplay between seasonal and water body perception. This needs more empirical accumulation (22). To address these identified research gaps—namely the insufficient attention to seasonal water body variations in northern cities, limited differentiation across water body types, and inadequate focus on youth populations—this study poses the following three research questions:

*Research Question 1*: What are the differential characteristics of restorative perception between waterfront and non-waterfront areas among Beijing youth?

*Research Question 2*: Do seasonal characteristics of northern cities—specifically, flowing water bodies in spring, summer, and autumn versus frozen water bodies in winter—influence youth restorative perception, and if so, how?

*Research Question 3*: What similarities and differences exist in youth perception characteristics across five water body types: lakes, wetlands, rivers, park water systems, and urban water features?

## 2 Methodology

### 2.1 Study area

Beijing was selected as the study city based on three primary considerations. Beijing is a representative Chinese megacity with a certain research value for urbanization processes and youth stress. The previous research indicated that urban green spaces and residents’ health perception in Beijing have a close relation (23). Being a city in the north, Beijing has distinct seasonal climatic characteristics. Moreover, the water freezes for almost 4 months in winter. As such, this provides a good background to see how seasonal change affects restorative perception. The results of previous studies revealed that Beijing’s green spaces have shown positive effects on the health of residents (24). Also, Beijing has rich water system resources consisting of various water bodies such as lakes, wetlands, and rivers.

The research study looked at 45 waterfront sites across five types of water bodies. The 45 waterfront sites encompassed eight lakes (e.g., Kunming Lake, Beihai Lake, Shichahai Lake), eight wetlands (e.g., Yeyahu Wetland, Hanshiqiao Wetland), 10 rivers (e.g., Yongding River, Liangma River), 10 park water systems (e.g., Olympic Forest Park, Chaoyang Park), and nine urban water features (e.g., Sanlitun Taikoo Li, Solana). Complete site inventories are provided in Appendix A.

**Figure 1:**
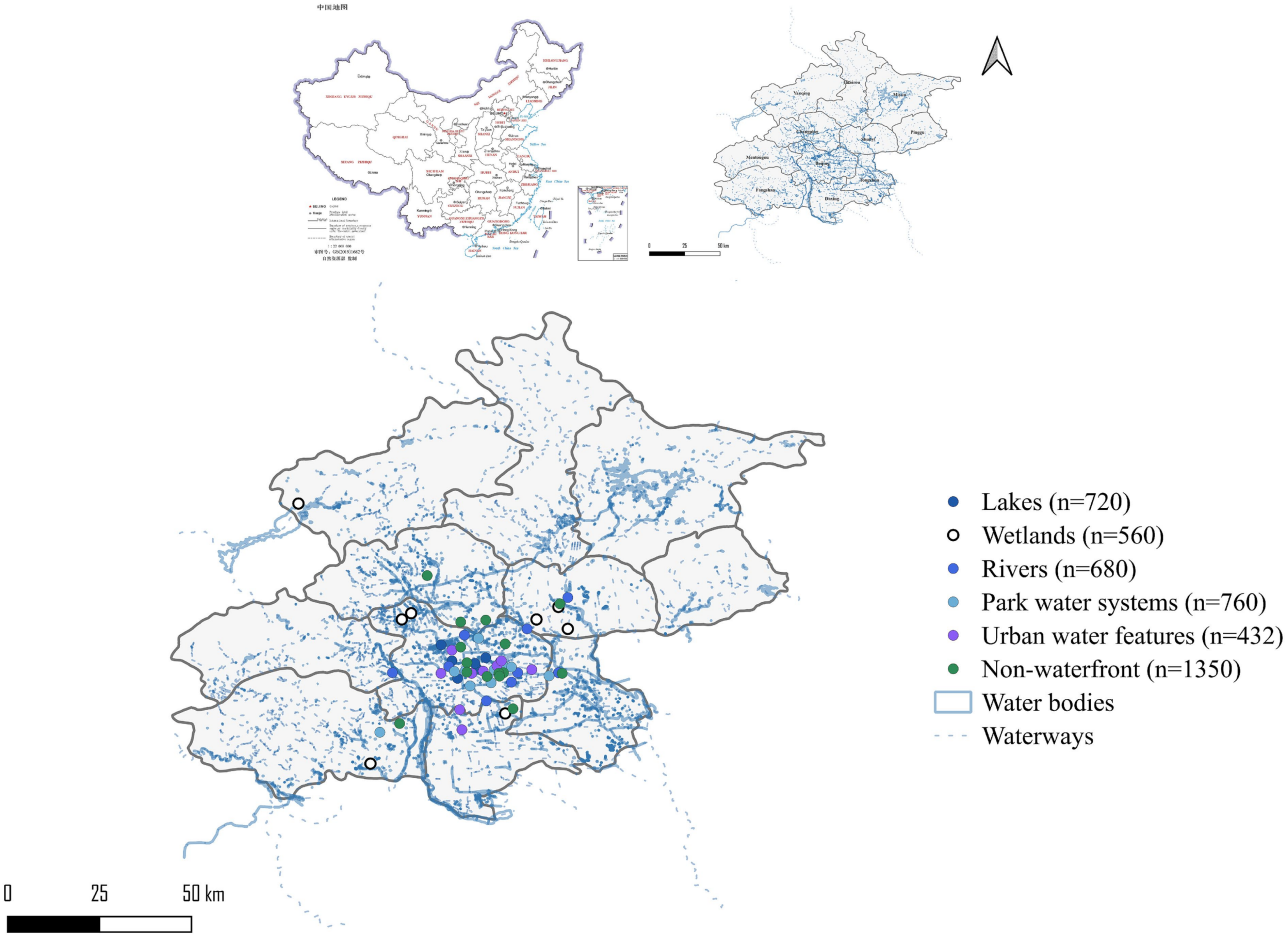
Spatial distribution of five water body types in Beijing.

The site selection is based on three principles. The site is spatially balanced, typologically representative and accessible. The three principles ensure coverage of the eastern, western, southern, northern and central districts of Beijing without spatial clustering. Fifteen non-waterfront control areas were established in major commercial and recreational districts to ensure comparability with waterfront sites. Control area selection followed systematic matching criteria: (1) analogous urban function (public recreational or commercial space, excluding residential-only areas); (2) comparable transit accessibility (all sites within 500 m of metro stations); and (3) similar social media posting activity levels (verified through preliminary platform searches). These matching criteria served to isolate the effect of water presence from potential confounding factors including urban function, accessibility, and visitor volume. Table A3 in Appendix A presents detailed comparability characteristics. A 50-meter buffer zone was employed to distinguish areas with direct waterfront access from non-directly accessible areas. This threshold was selected based on two considerations: first, it represents the immediate sensory exposure distance where visual, auditory, and olfactory waterfront stimuli remain directly perceptible; second, it aligns with the spatial granularity inherent in geotagged social media data. While the blue space exposure literature documents buffer distances ranging from 100 m to 1 km for residential proximity studies (5), our 50 m threshold captures immediate in-situ experience rather than residential proximity, appropriate for analyzing perceptions derived from geotagged posts. To assess robustness, sensitivity analyses were conducted using 30 m, 100 m, and 300 m buffer thresholds (see Results section). As illustrated in Figure 1, study sites were distributed across Beijing’s five principal districts (Dongcheng, Xicheng, Chaoyang, Haidian, and Fengtai) and extended suburban areas, ensuring comprehensive spatial coverage without clustering. Lakes (solid blue circles, *n* = 8) and wetlands (hollow circles, *n* = 8) were predominantly located in suburban green belts, while park water systems (green circles, *n* = 10) and urban water features (purple circles, *n* = 9) concentrated within central urban districts. Non-waterfront control areas (gray circles, *n* = 15) were matched to commercial districts across analogous spatial zones.

### 2.2 Data sources

Two platforms were selected for the collection of data; Douyin and Xiaohongshu were selected because of the high activity level of Chinese youth on these 2 platforms. Also, both these platforms have sophisticated geotagging functionalities. Social media data are proven to be an effective tool in assessing how the public perceive the accessibility, usability, and attractiveness of urban parks (25), thus providing new methodological avenues for research on environmental perception.

The data was collected over four complete seasons from March 2024 to February 2025. The collection procedure consisted of three approaches: filtering geotagged images obtained using points of interest located within the study areas, keyword searching using the terms lakeside, riverside, and other similar terms related to water environments, and the identification of the user age characteristics. The process of cleaning the data involves eliminating content of ads and promotions, removing duplicate posts, and removing samples with unclear geolocation information.

**Figure 2:**
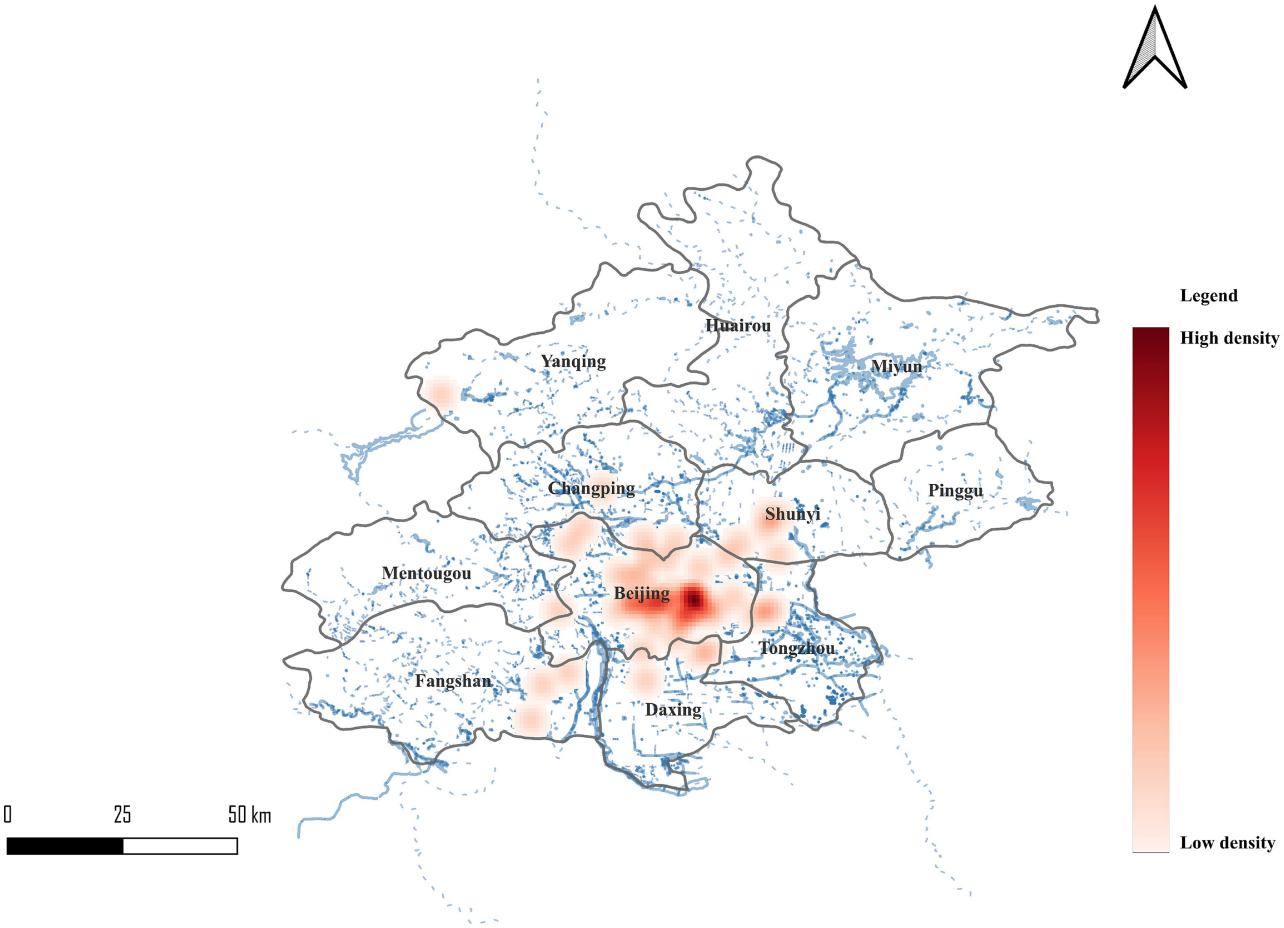
Spatial density of social media data in Beijing.

The final dataset comprised 4,502 valid entries. Information on the waterfront area included 3,152 entries which accounted for 70.0% of the entire sample. The entries comprised 720 for lakes, 560 for wetlands, 680 for rivers, and 760 entries for water systems in the park. In addition, there were 432 entries of water systems in the urban region. Non-waterfront control area data was covered by 1,350 entries or 30.0%. Kernel density analysis of social media data (Figure 2) reveals concentrated posting activity across three principal zones: the Central Urban District (highest density, depicted in red), the Tongzhou sub-center (moderate-high density), and dispersed suburban nodes surrounding major wetlands. This distribution pattern reflects both population density and waterfront accessibility, with moderate density observed in suburban areas indicating adequate data coverage beyond urban core concentrations. Over-all the urban area density zone is more concentrated in the Central Urban District and the Tongzhou District. The suburban area has moderate density distribution. And the data collection has a desirable spatial coverage.

### 2.3 Youth definition and identification

This study operationally defines “youth” as individuals aged 18–35 years, consistent with established definitions of Chinese millennials and young adults in demographic research (1). This age range encompasses late adolescents who have attained legal adulthood and young working professionals, representing the primary demographic experiencing urbanization-related psychological stress.

Youth identification on Douyin and Xiaohongshu proceeded through a three-step filtering protocol. First, profile-based screening identified users with publicly visible age indicators, including birth year in username, age disclosed in biographical fields, or student status indicators. Second, content-based inference was applied to users without explicit age disclosure, evaluating references to student life or early-career professional experiences, contemporary youth cultural references and linguistic patterns, and visual content suggesting young adult demographics. Third, conservative exclusion removed users whose age could not be reasonably verified, along with commercial accounts and promotional content.

Of the initial 6,074 posts collected, 1,572 (25.9%) were excluded due to: unverifiable age (*n* = 987, 16.2%), commercial or promotional content (*n* = 389, 6.4%), and ambiguous geolocation information (*n* = 196, 3.2%). The final sample of 4,502 entries represents users with reasonable confidence of youth status. A detailed screening flowchart is provided in Appendix B.

### 2.4 Ethics statement

This study utilized publicly available social media data from Douyin and Xiaohongshu platforms. Only publicly shared posts with geotagging were collected; private accounts and restricted content were not accessed. All usernames, profile information, and identifying details were removed during data processing, with only anonymized text content and geographic coordinates retained for analysis. As the study analyzed existing public content without direct participant interaction, formal ethics approval was not required under Chinese research regulations governing secondary analysis of publicly available information. The research protocol was reviewed by the institutional research committee for compliance with data privacy principles.

## 3 Data analysis

The qualitative research paradigm was adopted in this study by utilizing social media text analysis in data collection. Using NVivo software, three-level thematic coding was conducted following established qualitative research protocols (26). This approach proceeds through three sequential stages: open coding (line-by-line identification of initial concepts from raw text), axial coding (establishing relational linkages among emergent concepts), and selective coding (integrating concepts into overarching thematic categories) (27). The coding proceeded through three stages: open which coded concepts out of the raw text, axial which established relations between those concepts and selective which coded the main themes. This approach finally resulted in a thematic framework for stress recovery perception.

Two levels of triangulation strategies were used for credibility. The data were obtained from Douyin and Xiaohongshu at the data triangulation level, along with field observations. Non-participatory observations were conducted at representative waterfront spaces to document youth behavioral patterns, duration of stay, activity types, and seasonal variations. In the researcher triangulation level, two researchers conducted coding independently which is extensively used in social media research on environmental perception (28). The Cohen’s kappa coefficient for inter-coder agreement was 0.81, indicating an “almost perfect agreement.” Discrepant codes were resolved through discussion until consensus was achieved.

Using point-of-interest data with buffer zone analysis established the spatial attribution of data. According to the climatic characteristics of Beijing, spring is defined as March/May, summer as June/August, autumn as September/November, and winter as December/February.

## 4 Results

### 4.1 Data overview

A total of 4,502 valid data entries were collected across 60 study sites, comprising 45 waterfront locations and 15 non-waterfront control areas. Regarding platform distribution, Xiaohongshu contributed 2,557 entries at 56.8%, while Douyin contributed 1,945 entries at 43.2%. This dual-platform collection strategy ensured diversity in data sources. Spatial distribution exhibited waterfront area predominance, with waterfront samples numbering 3,152 entries at 70.0% and non-waterfront control area samples totaling 1,350 entries at 30.0%, thereby providing sufficient data support for comparative analysis between waterfront and non-waterfront environments.

Seasonal distribution demonstrated relative balance across all four seasons. Summer yielded the largest sample at 1,235 entries representing 27.4%, followed by autumn with 1,135 entries at 25.2% and spring with 1,094 entries at 24.3%. Winter samples were fewest yet remained at a reasonable level with 1,038 entries constituting 23.1%. This distribution pattern reflects seasonal preferences for waterfront activities among youth while ensuring adequate analytical samples for each season. Among the five water body types, park water systems generated the largest sample at 760 entries representing 16.9%, followed by lakes with 720 entries at 16.0% and rivers with 680 entries at 15.1%. Wetlands and urban water features yielded comparatively fewer samples at 560 entries representing 12.4% and 432 entries at 9.6%, respectively. As shown in Table 1, data collection achieved balanced coverage across dimensions of platform source, seasonal cycle, and water body type, establishing a solid foundation for subsequent analysis of the three research questions.

**Table 1:** Data collection and sample characteristics.

Statistical category	Item	Count	Percentage
Overall sample	Total valid entries	4,502	100%
	Waterfront samples	3,152	70.0%
	Non-waterfront samples	1,350	30.0%
Study sites	Waterfront sites	45	—
	Non-waterfront control areas	15	—
	Total sites	60	—
Water body type	Lakes (8 sites)	720	16.0%
	Wetlands (8 sites)	560	12.4%
	Rivers (10 sites)	680	15.1%
	Park water systems (10 sites)	760	16.9%
	Urban water features (9 sites)	432	9.6%
	Non-waterfront areas (15 sites)	1,350	30.0%
Platform distribution	Xiaohongshu	2,557	56.8%
	Douyin	1,945	43.2%
Seasonal distribution	Spring (Mar–May)	1,094	24.3%
	Summer (Jun–Aug)	1,235	27.4%
	Autumn (Sep–Nov)	1,135	25.2%
	Winter (Dec–Feb)	1,038	23.1%

### 4.2 Waterfront versus non-waterfront restorative perception

Prior to presenting comparative results, the coding procedures require methodological clarification. Each post could receive multiple thematic codes across dimensions (e.g., a single post coded for both sensory experience and emotional state). Theme proportions represent the percentage of total posts (*N* = 4,502) receiving each respective code; consequently, proportions do not sum to 100%. Reported ratios (e.g., “3.05-fold”) constitute descriptive comparisons of coding proportions rather than statistically inferred effect sizes.

To address the first research question, three-level NVivo coding was applied to systematically analyze social media texts from waterfront and non-waterfront areas. This analysis yielded a restorative perception thematic framework encompassing four dimensions: sensory experience, emotional state, activity behavior, and cognitive evaluation. Coding results revealed that waterfront areas exhibited higher restorative perception expressions across all dimensions compared to non-waterfront areas, though the magnitude of differences varied considerably across dimensions.

The sensory experience dimension displayed the most pronounced disparity. Sensory experience themes accounted for 25.7% in waterfront areas versus merely 8.4% in non-waterfront areas, representing a 3.05-fold difference. To assess the stability of this finding, robustness checks were conducted across multiple analytical conditions (Table 2).

**Table 2:** Robustness checks for waterfront versus non-waterfront comparisons.

Analysis type	Condition	Sensory experience ratio	Conclusion
Primary analysis	50 m buffer	3.05	Baseline
Buffer sensitivity	30 m	3.21	Stable
Buffer sensitivity	100 m	2.94	Stable
Buffer sensitivity	300 m	2.91	Stable
Platform stratification	Xiaohongshu	3.12	Stable
Platform stratification	Douyin	2.96	Stable

All sensitivity analyses yielded ratios within the 2.91–3.21 range, supporting the stability of primary findings. Buffer distance sensitivity tests confirm that the waterfront advantage persists across spatial thresholds commonly employed in blue space research. Additional checks restricting analysis to single-coded posts only (*n* = 2,847) yielded a ratio of 2.89, confirming results are robust to alternative counting conventions.

#### 4.2.1 Supplementary analysis: post type stratification

To assess whether findings were driven by superficial “check-in” posts versus substantive experiential content, the sample was stratified based on content characteristics. Posts were classified as check-in/sharing posts (*n* = 1,847, 41.0%), characterized by brief captions, photo-centric content, and hashtag prevalence, versus experience/reflection posts (*n* = 2,655, 59.0%), featuring detailed environmental descriptions and emotional expressions.

Comparative analysis revealed consistent patterns across both strata. Sensory experience themes appeared in 24.1% of check-in posts versus 26.8% of experience posts (difference within sampling variability). The waterfront advantage in sensory experience persisted across both categories (check-in: 2.89-fold; experience: 3.12-fold), supporting the robustness of primary findings against content-type bias.

#### 4.2.2 Location distribution and hotspot assessment

To preclude results being dominated by a limited number of highly frequented locations, post distribution across sites was examined. No single site contributed more than 5% of total posts (maximum: 4.2% at Olympic Forest Park), with median per-site contribution of 72 posts (IQR: 48–108). Given this relatively uniform distribution, no capping or weighting procedures were applied. However, sensitivity analyses excluding the five most-posted sites (*n* = 892 posts removed) confirmed that all primary findings remained stable (waterfront sensory advantage: 2.98-fold versus 3.05-fold in the full sample), indicating that conclusions are not driven by location-specific hotspots.

This finding suggests that multisensory stimulation provided by aquatic environments—encompassing visual, auditory, and tactile elements—is strongly associated with restorative experiences among youth. The activity behavior dimension exhibited substantial divergence as well, with waterfront areas at 24.1% approximately 1.94 times that of non-waterfront areas at 12.5%, suggesting that waterfront spaces are associated with higher recreational activity participation among youth. Differences in the emotional state dimension were relatively modest, with waterfront areas at 29.4% and non-waterfront areas at 26.4% yielding a ratio of 1.11, suggesting that emotional expression possesses certain universality in social media texts. Notably, the cognitive evaluation dimension accounted for 18.6% in waterfront areas while remaining virtually absent in non-waterfront areas, suggesting that waterfront environments are more frequently associated with positive reflections on spatial value among youth. Among specific restorative characteristics, calmness was particularly prominent. This theme accounted for 7.7% in waterfront areas while being completely absent in non-waterfront areas, revealing the distinctive value of aquatic environments in creating tranquil atmospheres.

**Figure 3:**
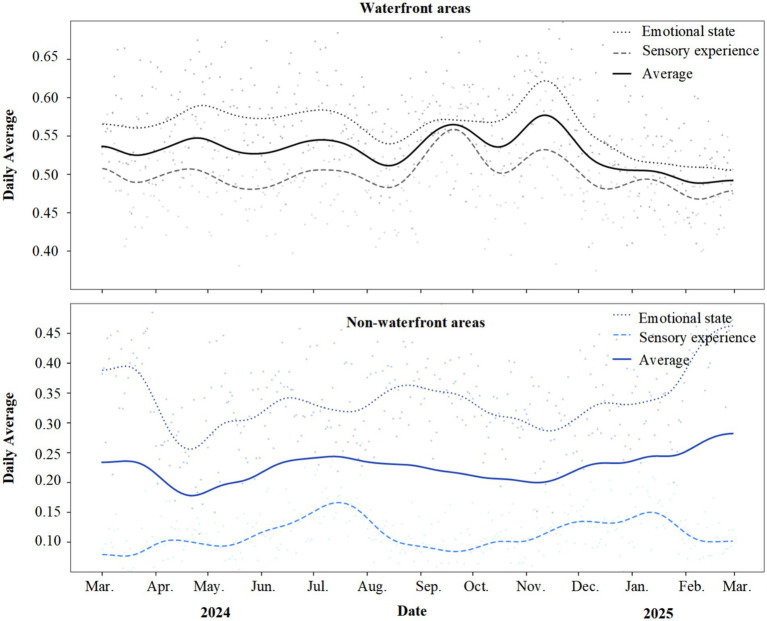
Annual trends in restorative themes: waterfront versus non-waterfront areas.

Annual trend analysis (Figure 3) demonstrates that restorative perceptions in waterfront areas consistently exceeded non-waterfront areas throughout the year, with the most pronounced disparity observed in the sensory experience dimension. As illustrated, these differences remained stable throughout the annual cycle. Emotional state and sensory experience curves for waterfront areas consistently maintained elevated levels, with daily mean values fluctuating within ranges of 0.55–0.62 and 0.48–0.53, respectively. Corresponding curves for non-waterfront areas were markedly lower, with emotional state fluctuating between 0.30 and 0.40 and sensory experience remaining at low levels between 0.08 and 0.17. The sensory experience gap between the two area types was particularly striking, with waterfront and non-waterfront curves forming a distinct separation band. Temporal analysis revealed that restorative perception in waterfront areas peaked during summer and autumn from June through November, declined during winter from December through February, yet remained significantly higher than non-waterfront levels throughout the year. This trend provided preliminary insights for subsequent seasonal difference analysis.

### 4.3 Seasonal variations in restorative perception

To address the second research question, analysis focused on how Beijing’s seasonal characteristics as a northern city influence youth waterfront restorative experiences. The winter freezing period for Beijing’s water bodies extends approximately 4 months, and this seasonal transformation in physical form provides a unique context for examining restorative differences between flowing and frozen water bodies. Coding results demonstrated significant differences in restorative perception between flowing water seasons spanning spring, summer, and autumn versus the frozen water season of winter.

The sensory experience dimension remained relatively stable across spring, summer, and autumn at 26.1, 25.8, and 27.2%, respectively. The emotional state dimension similarly maintained elevated levels across these three seasons at 29.7, 30.6, and 30.2%. Upon entering winter, traditional sensory experience theme proportions declined to 23.4% and emotional state decreased to 26.7%, reflecting the attenuating effect of water body freezing on conventional restorative experiences.

**Figure 4:**
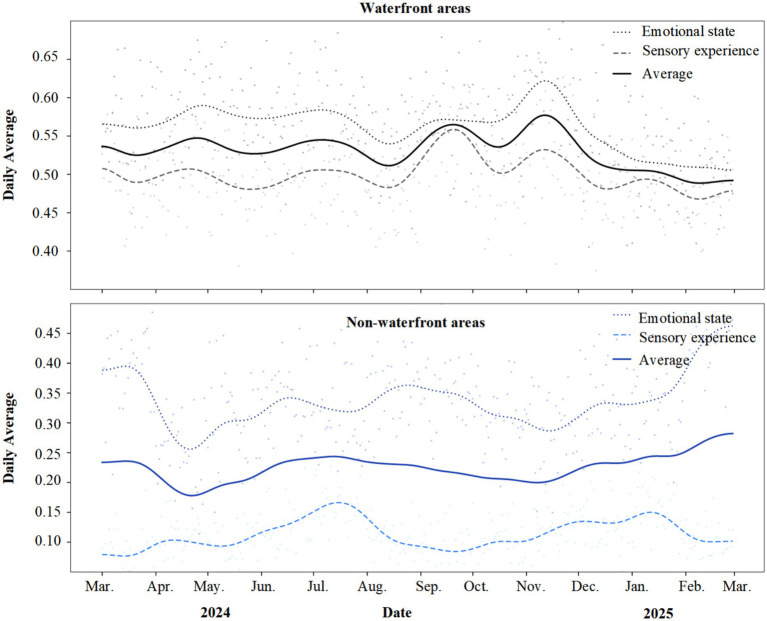
Seasonal variation in restorative themes across water body types.

Seasonal variation analysis (Figure 4) reveals cyclical patterns across water body types, with perceptions peaking during summer months and declining in winter, though never approaching baseline levels observed in non-waterfront areas. As depicted, lakes, wetlands, and rivers exhibited comparable seasonal fluctuation patterns throughout the annual cycle. During spring and summer from March through August, restorative perception curves for all three natural water body types maintained elevated levels. Lakes reached peak values exceeding 0.70 in daily means during August and September. Entering winter from December through February, curves for all water body types displayed evident downward trends, with lakes showing the most substantial decline from peak values of 0.70 to approximately 0.45. Notably, winter curves declined but did not approach zero, suggesting that frozen water bodies retain certain restorative value.

Further analysis revealed that winter waterfront experiences exhibited “alternative restoration” characteristics. The ice landscape theme accounted for 5.8%, with youth expressing positive aesthetic experiences regarding frozen lake surfaces and snow-reflected scenery. The seasonal rituals theme represented 3.9%, as seasonally distinctive waterfront activities such as winter solstice lake viewing and New Year blessings emerged as unique forms of youth stress relief. These findings suggest that winter water bodies in northern cities do not represent a “blank period” for restorative functions but rather appear to maintain psychological adjustment value through alternative pathways.

### 4.4 Restorative perception across water body types

To address the third research question, restorative perception characteristics were systematically compared across five water body types: lakes, wetlands, rivers, park water systems, and urban water features. Coding results revealed distinctly differentiated patterns in restorative theme composition, with each water body type forming a unique functional positioning.

Lakes were dominated by tranquility and calmness characteristics. The emotion-calmness theme accounted for 11.6% and sensory experience for 11.5%, both representing the highest values among the five water body types and embodying the distinctive advantages of lake environments in creating serene atmospheres. Wetlands were distinguished by unique ecological value, with the cognition-unique value theme reaching 12.2%, reflecting youth cognitive appreciation of wetland ecosystems. Rivers emphasized dynamic experiences and vitality. The emotion-relaxation theme at 10.0% ranked highest among the five types, with sensory experience at 9.8%. Water flow sounds and dynamic landscapes constituted core attractions of river environments.

Park water systems and urban water features exhibited stronger social function attributes. Park water systems highlighted convenience and social functions, with the activity-social theme at 8.9% and cognition-accessibility at 9.0%, embodying the accessibility advantages of urban parks as everyday recreational spaces. Urban water features displayed the most distinctive characteristics. The activity-social/sharing theme reached 14.6% and cognition-accessibility 12.7%, both significantly exceeding other water body types. This pattern indicates that water features in commercial spaces have become important venues for youth social media check-ins and daily recreation. As presented in Table 3, the five water body types formed a functional spectrum ranging from “tranquil aesthetics” to “social sharing” in restorative theme composition. This functional spectrum across water body types represents a novel empirical contribution of this research, providing differentiated evidence for urban waterfront planning that transcends treating all blue spaces as functionally equivalent. Natural water bodies including lakes, wetlands, and rivers emphasized sensory and emotional restoration experiences, while artificial or semi-artificial water bodies comprising park water systems and urban water features predominantly served social interaction and convenient accessibility functions.

**Table 3:** Restorative theme distribution across area types and water body categories.

Perception dimension	WF	Non-WF	Lakes	Wetlands	Rivers	Park	Urban	Seasonal
Sensory experience	25.7%	8.4%	11.5%*	7.3%	9.8%	—	9.2%	Autumn 27.2%
Emotion-relaxation	29.4%	26.4%	11.5%	8.2%	10.0%*	9.6%	7.5%	Summer 30.6%
Emotion-calmness	7.7%	0.0%	11.6%*	8.6%	—	6.7%	—	—
Activity-social/sharing	24.1%	12.5%	5.3%	—	8.6%	8.9%*	14.6%*	—
Cognition-accessibility	18.6%	0.0%	—	—	—	9.0%	12.7%*	—
Cognition-unique value	—	—	—	12.2%*	—	—	—	—
Winter-specific	—	—	—	—	—	—	—	Ice 5.8%

*Indicates the most prominent characteristic for each water body type; “—” indicates proportion below 5% or theme not prominently observed. Percentages represent the proportion of posts coded with respective themes; as posts may receive multiple codes, percentages do not sum to 100%. The Seasonal Peak column indicates the season with highest expression for each dimension in waterfront areas.

### 4.5 Thematic framework of restorative experiences

Synthesizing the preceding analyses, three-level NVivo coding was employed to construct an overarching thematic framework for youth waterfront restorative experiences. As shown in Table 4, the coding framework encompasses four core dimensions—sensory experience, emotional state, activity behavior, and cognitive evaluation—plus a winter-specific dimension. This framework yielded five first-level codes through selective coding, 18 s-level codes through axial coding, and numerous third-level codes through open coding.

**Table 4:** NVivo three-level coding framework.

First-level code (selective coding)	Second-level code (axial coding)	Third-level code (open coding)	Representative expressions
Sensory experience	Visual perception	Water reflections, shimmering light, open views	“The shimmering lake surface is so beautiful”
	Auditory perception	Flowing water, birdsong, wind sounds	“Listening to water sounds is so stress-relieving”
	Tactile perception	Gentle breeze, warm sunlight, cool mist	“The lakeside breeze feels so comfortable”
	Olfactory perception	Fresh air, floral fragrance	“The air is exceptionally fresh”
	Multisensory perception	Integrated sensory experiences	“All my senses feel healed”
Emotional state	Relaxation	Physical and mental relaxation, fatigue relief	“I feel completely relaxed”
	Calmness	Inner peace, tranquil mood	“My mind instantly became calm”
	Pleasure	Happiness, joyful mood	“My mood has improved so much”
	Stress relief	Decompression, releasing pressure	“All the stress is gone”
Activity behavior	Static activities	Daydreaming, watching scenery, sunbathing, reading	“Sitting by the lake in a daze”
	Dynamic activities	Walking, jogging, cycling, boating	“Walking along the river is so pleasant”
	Social activities	Dating, gatherings, family activities	“Having a picnic with friends”
	Recording/sharing	Photography, check-ins, social media posts	“Great spot for photos”
Cognitive evaluation	Spatial quality	Clean environment, complete facilities	“The environment is well maintained”
	Accessibility	Convenient transportation, good location	“Direct metro access, very convenient”
	Unique value	Ecological value, scarcity	“Rare nature within the city”
	Seasonal experience	Four-season changes, seasonal landscapes	“The spring cherry blossoms are beautiful”
Winter-specific	Ice landscape	Ice surface, snow, silver-white scenery	“The mirror-like ice surface is stunning”
	Seasonal rituals	Winter exclusives, seasonal ceremonies	“Coming here in winter feels ceremonial”

Second-level codes within each dimension were clearly delineated. Sensory experience comprised visual perception, auditory perception, tactile perception, olfactory perception, and multisensory perception. Emotional state encompassed relaxation, calmness, pleasure, and stress relief. Activity behavior was divided into static activities, dynamic activities, social activities, and recording/sharing. Cognitive evaluation included spatial quality, accessibility, unique value, and seasonal experience. The winter-specific dimension contained two themes: ice landscape and seasonal rituals. Representative expressions such as “the shimmering lake surface is so beautiful,” “listening to water sounds is so stress-relieving,” and “I feel completely relaxed” authentically captured youth waterfront restorative experiences.

**Figure 5:**
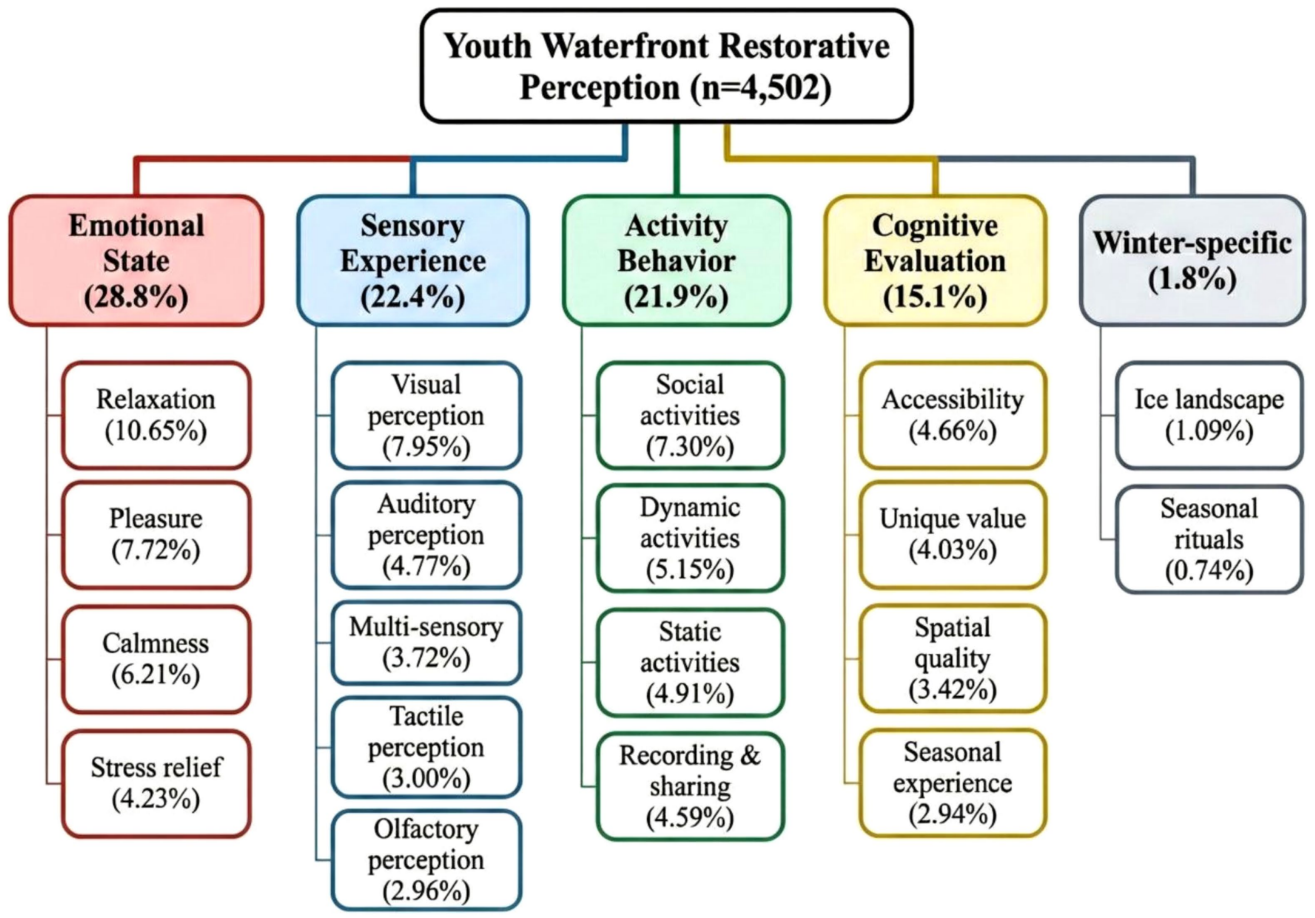
Youth waterfront restorative perception thematic map.

As illustrated in Figure 5, the five dimensions displayed differentiated proportional distributions within the overall thematic framework. The emotional state dimension accounted for the highest proportion at 28.8%, with relaxation at 10.65% representing the most dominant second-level theme. Sensory experience at 22.4% and activity behavior at 21.9% showed comparable proportions, dominated by visual perception at 7.95% and social activities at 7.30%, respectively. Cognitive evaluation accounted for 15.1%, with accessibility at 4.66% as the leading theme within this dimension. Although the winter-specific dimension represented a modest proportion at 1.8%, the emergence of ice landscape at 1.09% and seasonal rituals at 0.74% validated the existence of “alternative restoration” in northern cities.

Regarding research reliability, inter-coder agreement measured by Cohen’s Kappa coefficient reached 0.81, indicating “almost perfect agreement.” Dual-coded samples totaled 675 entries representing 15.0% of the total sample. Initial agreement rate stood at 83.1%, improving to 97.2% following discussion and consensus, demonstrating high reliability of coding results.

## 5 Discussion

### 5.1 Theoretical implications

This study employed systematic social media text analysis to understand how Beijing youth perceive the stress-relieving functions of urban waterfront environments where all three research questions received empirical support. With reference to RQ1, waterfront areas were found to be significantly better off than non-waterfront areas with regard to the sensory experience dimension with a difference of 3.05 times. Moreover, calmness as a unique value of waterfront spaces was altogether absent in non-waterfront spaces. Similar findings had been documented in literature such as urban blue space regeneration and renewal as an effective public health intervention (29), positive effects of urban nature exposure on resident mental health (30), and substantial associations between use of urban parks and physical and mental wellbeing (31). The current research takes these conclusions into the domain of youth and waterfronts, affirming blue spaces as an important asset for youth stress relief. Concerning RQ2, the study reflects an “alternative restoration” phenomenon in the winter waterbodies of northern cities; while the familiar sensory experiences decline, the emergence of different themes like Ice Landscape at 5.8% and Seasonal Rituals at 3.9% reveals that frozen waterbodies do not represent a “blank period” for restorative functions. In the context of the RQ3, the five water body types formed a differentiated functional spectrum from “tranquil aesthetics” to “social sharing,” which provides empirical evidence to refine waterfront space planning.

The findings validate and extend Attention Restoration Theory and Stress Recovery Theory from a theoretical perspective. Specifically, the “alternative restoration” concept extends these theoretical frameworks by demonstrating that psychological restoration can occur not only through conventional natural landscapes but also through physically transformed environmental forms. Frozen waterscapes elicit “fascination” (per ART) via novel ice aesthetics rather than flowing water dynamics, while seasonal rituals facilitate “being away” through cultural meaning-making rather than physical displacement from routine environments. The findings affirm ART’s core principles that contain fascination and being away, which are characteristics of restorative environments. Thus, it appears that multi-sensory stimuli from shimmering surfaces of water and sounds of flowing water are environmental elements that captivate involuntary attention, allowing youth to disengage from daily stressful contexts for a time. The dimension of emotional state was the most represented in the thematic structure at 28.8%. The presence of themes like relaxation at 10.65% and stress relief at 4.23% suggests that SRT works because natural settings evoke positive emotions and alleviate physiological stress. Studies reveal that mental health problems faced by the contemporary youth are becoming a global issue. The present study confirms that waterfront environments can be important environmental resources for youth stress coping. According to past literature (32), green spaces affect mental health of university students via mediating impact of solitude capacity and perceived restoration. The present research further shows that blue spaces, too, possess restorative mediating impacts. Calmness is a unique contribution of water bodies and has not been highlighted as much in green space research.

The “alternative restoration” hypothesis adds a seasonal dimension to ART and SRT as restoration environmental characteristics may change functionally as their physical forms change.

To delineate conceptual boundaries, “alternative restoration” refers to restorative effects occurring when natural environments undergo substantial physical transformation (such as water body freezing) while retaining psychological benefits through mechanistically shifted pathways. Three necessary conditions bound this concept: (1) the environment possesses established restorative value in its primary form; (2) transformation substantially alters sensory characteristics; and (3) users continue reporting positive psychological experiences through alternative pathways.

**Figure 6:**
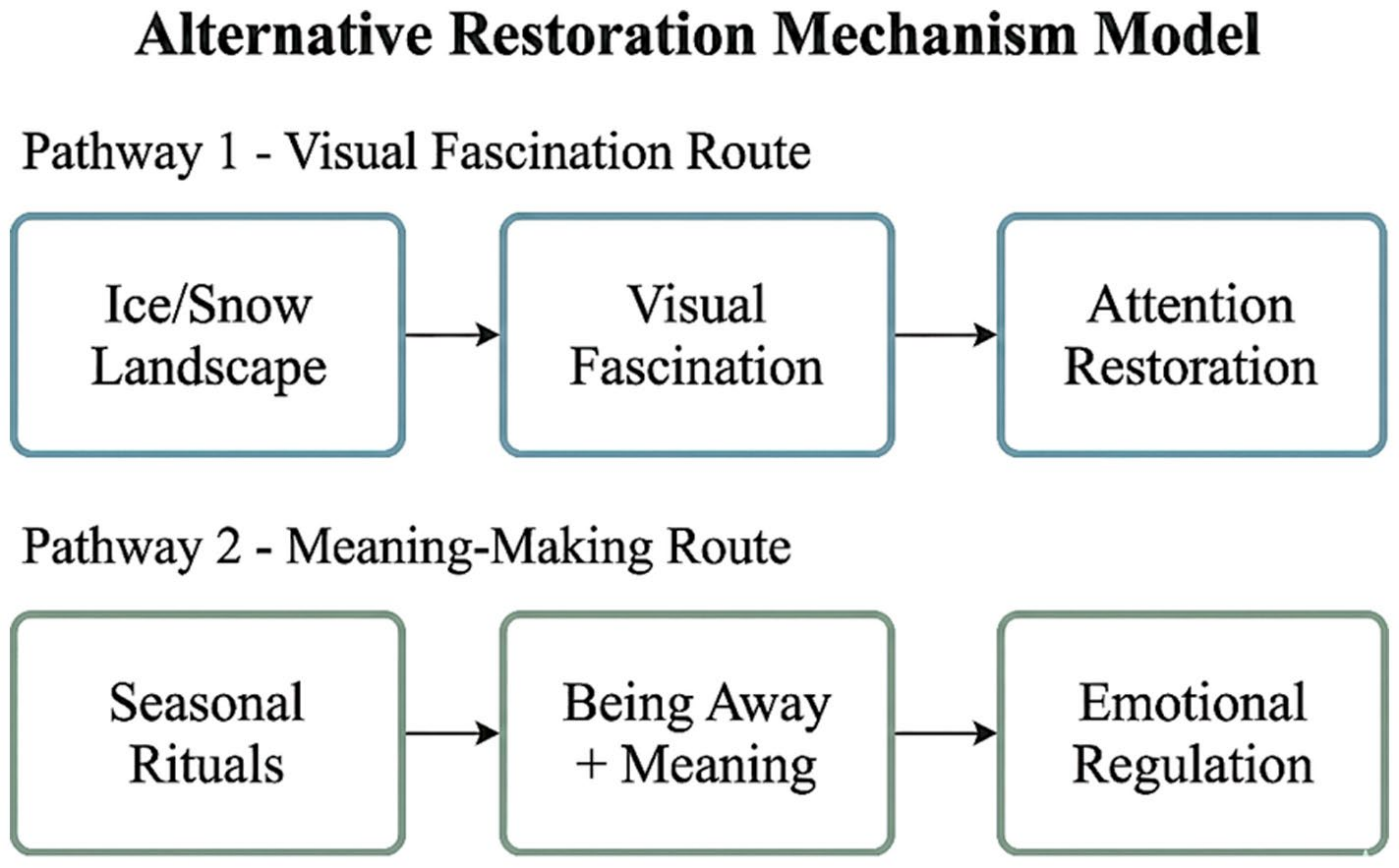
Alternative restoration mechanism model for winter waterscapes. Two pathways through which frozen water bodies provide restoration when conventional blue space mechanisms are unavailable.

This formulation differs from prior winter restoration research. Studies examining winter forests (33) and cold-season nature interventions (34) documented that winter environments retain restorative benefits, yet did not specify alternative mechanisms when primary environmental features undergo transformation. The present contribution identifies two distinct pathways operative when conventional water-related stimuli are absent (Figure 6): visual fascination through ice aesthetics, and meaning-making through seasonal rituals.

Representative expressions illustrating these pathways include:

Ice landscape pathway: The mirror-like ice surface is stunning; standing by the lake, I felt completely calm.

Seasonal ritual pathway: Coming to Shichahai specifically on Winter Solstice feels like a farewell ceremony for the year.

### 5.2 Practical implications

The research findings offer practical solutions, from two perspectives: seasonally adaptive design of the northern cities and differentiated development of water bodies.

### 5.3 Winter adaptive design

Waterfront space planning for cities in the north must take on board the transformation in the physical form of winter water bodies and the continuity of their restorative functions. The fact that our ice landscapes and seasonal ritual offer alternative pathways for the winter youth to relieve their stress resonates with an interesting finding from related literature. This assertion was made in light of the findings from related literature exploring the restorative potential of winter urban parks. This is in light of previous studies that showed how community parks can retain restorative functions when elaborate design specifications are incorporated, even in the winter months (35). Experience from the Nordic cities suggests that cold-climate cities can improve the health of their residents through resilient planning of outdoor recreation and green infrastructure (36). Consequently, winter waterfront design in northern cities should prioritize: (1) protecting and enhancing ice landscape visual appeal through strategic viewpoint placement and sightline management; (2) establishing demarcated safe zones for ice-related recreational activities; (3) designing waterfront festival spaces that reinforce seasonal ritual significance (e.g., winter solstice viewing platforms, illuminated ice zones); and (4) providing wind-sheltered warming facilities (e.g., heated rest stations, windbreak structures) to extend comfortable outdoor stay duration.

### 5.4 Differentiated planning based on water body type

Based on that current differentiated restorative functions of various waters, the five water body types need to be planned. Natural water elements like lakes, wetlands, rivers, and the like are characterized as the spaces for the users who prefer sensory and emotional experiences. Moreover, the planning of natural water has to protect the natural character and ecological value. Further, the planning should facilitate the creation of quiet, low-disturbing spatial atmospheres. The park water systems and urban water features constitute artificial or semi-artificial water bodies. They mainly fulfill social interaction and convenience of accessibility functions. First, prior research examined the health-related outcomes of artificial water feature interactions systematically (37). Second, findings confirm that urban water features have become key venues for youth social media engagement and quotidian recreation. Design specifications for artificial water bodies should incorporate visually distinctive landscape nodes—such as signature water features, photogenic seating installations, and architecturally notable fountain displays—to support the recording and sharing behaviors characteristic of contemporary youth engagement patterns. Studies on the relationship between blue space in a neighborhood and health of residents signify that access is a major factor affecting health benefits of blue space (38). The above finding that park water systems and urban water features performed very well on access themes supports this view. It is suggested that blue spaces be integrated into youth mental health policy frameworks for urban areas. Regarding waterfront space planning, it is best to include youth-friendly functional configurations. For example, quiet corners that can be used for solitary meditation, open spaces suitable for social activities and landscape nodes. The landscape nodes help with recording and sharing. In addition, year-round waterfront space use guidance mechanisms should be included as well.

### 5.5 Limitations and future directions

Several limitations of this study warrant acknowledgment. The data from social media display “representativeness bias.” This means that the sample is tilted toward the active user group. Thus, the waterfront experience of silent users and non-social media users was not captured. A more structurally significant concern is the platform-specific nature of positive bias on Xiaohongshu and Douyin. Unlike general-purpose platforms such as Twitter, these applications function as curated self-presentation ecosystems in which content production is inseparable from identity performance and social approval-seeking. Users do not merely report experiences; they selectively construct and publish representations of experiences deemed aesthetically or emotionally worthy of public display (17). This dynamic operates as a systematic filter rather than random noise, meaning that negative or affectively neutral waterfront encounters are structurally absent from the dataset rather than randomly underrepresented. The consequence for interpretation is non-trivial: the observed coding proportions—including the 3.05-fold sensory experience ratio and the 7.7% calmness theme—reflect the frequency of positively framed public expressions among active users, not the distribution of restorative experiences across the youth population as a whole. The magnitude of these effects should therefore be understood as an upper-bound estimate of the population-level phenomenon. Prior comparative studies have demonstrated that social media-derived perceptions of urban green spaces diverge systematically from survey-based assessments, with platform-specific positive framing producing higher satisfaction scores than representative sampling methods (39). Future mixed-methods research integrating physiological indicators such as heart rate variability or skin conductance with social media text analysis would provide the calibration necessary to establish where platform-amplified perception diverges from objectively measurable restoration. Furthermore, waterfront sites were predominantly recreational (78%) whereas control areas were predominantly commercial (87%), reflecting Beijing’s urban geography. Although this functional difference constitutes a potential confounder, the matching criteria employed—comparable transit accessibility and equivalent social media posting volumes—control for visitor flow and urban accessibility as confounding variables, yet cannot disentangle the contribution of water presence from the broader constellation of environmental affordances that co-occur with recreational waterfront settings. The observed waterfront advantage should accordingly be interpreted as the effect of waterfront environments as integrated spatial configurations rather than the isolated perceptual effect of water per se. The quality research paradigm is subjective by nature. The independent dual coding and triangulation strategies employed in this research notwithstanding, the extraction and interpretation of themes will inevitably be researcher-driven. The study’s geographic limitation to only Beijing means the conclusions cannot be generalized to other northern cities or wider nationwide setting. Due to the cross-sectional research design, we cannot establish causal claims regarding whether waterfront exposure produces sustained stress reduction, as the observed associations reflect momentary perceptual expressions rather than longitudinal psychological outcomes. Social media text captures expressed emotional responses at the moment of posting, which may not correspond to sustained psychological change or reflect the full affective trajectory of waterfront experience. Further research may expand on several fronts. Other northern cities (e.g., Harbin, Shenyang, Tianjin) may be selected for comparative research to test the cross-city stability of the alternative restoration hypothesis. The three-level NVivo coding framework developed in this study is theoretically anchored in ART and SRT, and its categorical structure—spanning sensory experience, emotional state, activity behavior, cognitive evaluation, and winter-specific dimensions—is derived from mechanisms rather than Beijing-specific cultural content, suggesting structural applicability to cities sharing comparable climatic and urbanization profiles. Cross-city application would nonetheless require contextual adaptation: local water body configurations, regional cultural practices surrounding seasonal rituals, and platform usage patterns among youth may introduce variation that warrants iterative refinement of second- and third-level codes while preserving the overarching framework architecture. Mixed methods may also be adopted. Social media analysis may be combined with questionnaire survey and/or physiological measurement to enhance the generalisability and evidential strength of research findings. Longitudinal research may track the developmental trajectory of youth waterfront experience. Finally, the theoretical exploration of winter frozen water body restoration mechanism may also be deepened for further clarification of psychological processes and boundary conditions of alternative restoration. Future research could extend to visual data analysis of posted photographs and videos, employing image recognition techniques to extract environmental features and correlate them with textual restoration expressions.

## 6 Conclusion

Drawing on 4,502 geolocated social media entries from Douyin and Xiaohongshu platforms, this study employed three-level NVivo thematic coding and triangulation methods to systematically examine how Beijing youth perceive the stress-relief functions of urban waterfront environments, with all three research questions receiving empirical support. Regarding RQ1, waterfront areas demonstrated significant superiority over non-waterfront areas in restorative perception, with the sensory experience dimension exhibiting a 3.05-fold difference, and calmness as a distinctive value of waterfront environments was entirely absent in non-waterfront areas, indicating that aquatic environments provide irreplaceable stress-relief resources for youth. Regarding RQ2, winter water bodies in northern cities exhibited “alternative restoration” characteristics, and the emergence of distinctive themes including ice landscape at 5.8% and seasonal rituals at 3.9% demonstrates that frozen water bodies continue to exert psychological adjustment effects through alternative pathways. Regarding RQ3, the five water body types formed differentiated functional positions, with natural water bodies emphasizing tranquil aesthetics and sensory restoration while artificial water bodies predominantly serve social sharing and convenient accessibility functions. Theoretically, this research validates the applicability of Attention Restoration Theory and Stress Recovery Theory within the Chinese urban blue space context, and the proposed “alternative restoration” hypothesis enriches the seasonal dimension of restorative environment theory while addressing a gap in youth-focused research; methodologically, the integrated framework of social media text collection, three-level NVivo coding, and triangulation provides a replicable methodological pathway for environmental perception research; practically, the differentiated characteristics across the five water body types offer empirical evidence for refined planning of youth-friendly waterfront spaces. Despite acknowledged limitations—including representativeness bias, single-city geographic constraints, and causal inference restrictions inherent in cross-sectional designs—future research may address these through cross-city comparative studies and mixed-methods approaches. This study confirms that urban blue spaces, across diverse physical forms and seasonal conditions, constitute significant public health resources for youth psychological wellbeing in high-density urban environments. The finding that urban water features in commercial districts exhibit the highest rates of social sharing and accessibility-oriented coding carries a specific policy implication that extends beyond conventional park planning: it indicates that youth in high-stress urban contexts are already enacting spontaneous environmental micro-breaks within commercially embedded blue spaces, suggesting that the health-relevant behavior exists but lacks intentional infrastructural support. This behavioral pattern provides a concrete entry point for integrating blue space interventions into urban mental health policy—not solely through the expansion of dedicated waterfront parks, but through the deliberate incorporation of restorative water features into the everyday commercial and institutional environments that youth frequent under conditions of academic and occupational stress. Strategic planning of waterfront environments, encompassing winter functionality provisions in northern climates and restorative design standards for artificial water features in high-density commercial zones, warrants systematic integration into urban public health policy frameworks.

## Data Availability

The raw data supporting the conclusions of this article will be made available by the authors, without undue reservation.
