# Supplementary material for: Youth perceptions of urban waterfront environments for stress relief: a social media text analysis study in Beijing
Source: Front Public Health. 2026 Apr 7;14:1816233. doi: 10.3389/fpubh.2026.1816233 (PMC13096025; doi:10.3389/fpubh.2026.1816233)
Supplement: Supplementary file 1 [file Table_1.docx]

**Appendix A**

**Table A1.** Waterfront sites (N=45)

| **Water Body Type** | **Sites** |
| --- | --- |
| **Lakes (n=8)** | Kunming Lake, Beihai Lake, Shichahai Lake, Longtan Lake, Qingnian Lake, Lianhua Pond, Tuanjie Lake, Zizhuyuan Lake |
| **Wetlands (n=8)** | Yeyahu Wetland, Hanshiqiao Wetland, Nanhaizi Park, Wenyu River Wetland Park, Cuihu Wetland, Daoxianghu Wetland, Liulihe Wetland, Chaobai River Wetland |
| **Rivers (n=10)** | Yongding River, Liangma River, Qing River, Liangshui River, Tonghui River, North Canal, Chaobai River, Wenyu River, Kunyu River, Xiaotaihou River |
| **Park water systems (n=10)** | Olympic Forest Park, Chaoyang Park, Taoranting Park, Yuyuantan Park, Honglingjin Park, Longtan Park, Ritan Park, Yuetan Park, Qinglong Lake Park, Dongjiao Wetland Park |
| **Urban water features (n=9)** | Sanlitun Taikoo Li, Wangfujing, Xidan Joy City, Livat Shopping Center, Changying Tianjie, Solana, Zhongguancun, Wukesong Hi-Park, Longfor Paradise Walk Daxing |

**Table A2.** Non-waterfront control areas (N=15)

| **No.** | **Site Name** | **District** | **Primary Function** |
| --- | --- | --- | --- |
| 1 | Wangjing SOHO | Chaoyang | Commercial Office |
| 2 | Guomao CBD | Chaoyang | Commercial Office |
| 3 | Xizhimen | Xicheng | Transportation Hub |
| 4 | Chongwenmen | Dongcheng | Commercial Mixed |
| 5 | Wudaokou | Haidian | Commercial/Education |
| 6 | Dongzhimen | Dongcheng | Transportation Hub |
| 7 | Shuangjing | Chaoyang | Commercial Mixed |
| 8 | Jianguomen | Dongcheng | Commercial Office |
| 9 | Dawanglu | Chaoyang | Commercial Mixed |
| 10 | Sanyuanqiao | Chaoyang | Commercial Office |
| 11 | Liuliqiao | Fengtai | Transportation Hub |
| 12 | Fengtai Science Park | Fengtai | Technology Park |
| 13 | Yizhuang | Daxing | Technology Park |
| 14 | Huilongguan | Changping | Residential/Commercial |
| 15 | Tiantongyuan | Changping | Residential/Commercial |

**Table A3.** Comparability characteristics of waterfront and non-waterfront control areas

| **Characteristic** | **Waterfront (n=45)** | **Non-waterfront (n=15)** |
| --- | --- | --- |
| Mean metro accessibility | 320m | 280m |
| Mean social media post volume | 70 posts/site | 90 posts/site |
| Primary function type | Recreational/Park (78%) | Commercial/Mixed (87%) |

**
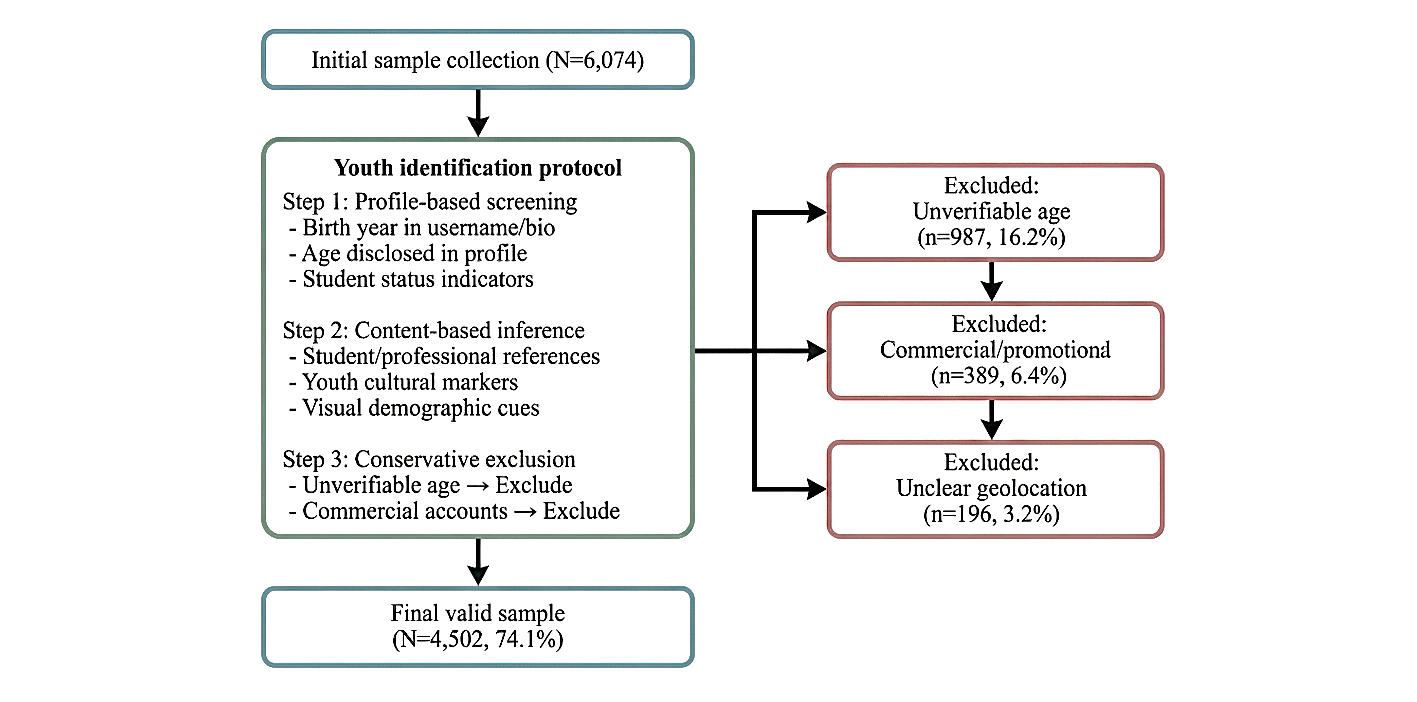
Appendix B**

**Figure B1.** Data screening and youth identification flowchart

**Table B1.** Youth identification decision rules

| **Evidence Type** | **Decision Rule** | **Confidence Level** |
| --- | --- | --- |
| Explicit birth year | 1989–2006 | High |
| Student status indicator | Current undergraduate/graduate | High |
| Early career indicator | 1–5 years post-graduation | Medium |
| Cultural marker inference | Youth subculture references | Low (requires corroboration) |

*Note:* Classification proceeded hierarchically. Explicit disclosure took precedence over inference. Posts from users meeting multiple criteria were assigned to the youngest applicable category. Visual assessment was used only as supplementary confirmation, never as sole criterion.
